# The Soliton and the Action Potential – Primary Elements Underlying Sentience

**DOI:** 10.3389/fphys.2018.00779

**Published:** 2018-06-25

**Authors:** Andrew S. Johnson, William Winlow

**Affiliations:** ^1^Independent Scientist, Villelongue de la Salanque, France; ^2^NPC Newton, Villelongue de la Salanque, France; ^3^Dipartimento di Biologia, Università degli Studi di Napoli, Naples, Italy; ^4^Institute of Ageing and Chronic Disease, University of Liverpool, Liverpool, United Kingdom; ^5^NPC Newton, Preston, United Kingdom

**Keywords:** sentience, action potentials, soliton, phase ternary computation, brain neural networks

## Abstract

At present the neurological basis of sentience is poorly understood and this problem is exacerbated by only a partial knowledge of how one of the primary elements of sentience, the action potential, actually works. This has consequences for our understanding of how communication within the brain and in artificial brain neural networks (BNNs). Reverse engineering models of brain activity assume processing works like a conventional binary computer and neglects speed of cognition, latencies, error in nerve conduction and the true dynamic structure of neural networks in the brain. Any model of nerve conduction that claims inspiration from nature must include these prerequisite parameters, but current western computer modeling of artificial BNNs assumes that the action potential is binary and binary mathematics has been assumed by force of popular acceptance to mediate computation in the brain. Here we present evidence that the action potential is a temporal compound ternary structure, described as the computational action potential (CAP). The CAP contains the refractory period, an analog third phase capable of phase-ternary computation via colliding action potentials. This would best fit a realistic BNN and provides a plausible mechanism to explain transmission, in preference to Cable Theory. The action potential pulse (APPulse), is made up of the action potential combined with a coupled synchronized soliton pressure pulse in the cell membrane. We describe a model of an ion channel in a membrane where a soliton deforms the channel sufficiently to destroy the electrostatic insulation thereby instigating a mechanical contraction across the membrane by electrostatic forces. Such a contraction has the effect of redistributing the force lengthways thereby increasing the volume of the ion channel in the membrane. Na ions, once attracted to the interior, balance the forces and the channel reforms to its original shape. A refractory period then occurs until the Na ions diffuse from the adjacent interior space. Finally, a computational model of the action potential (the CAP) is proposed with single action potentials significantly including the refractory period as a computational element capable of computation between colliding action potentials.

## Introduction

Sentience may be thought of as the highest ability to perceive events in the context of previous or future events, resulting in conscious non-reflex behavioral modification(s) and is dependent on self-awareness. Sentience must encompass elements of both time and complexity and is dependent upon individual experiences. The generation of sentience and other behavior must depend upon the brain’s ability at the level of neurons to compute nerve impulses according to timing defined by the biological processes present.

To understand how we compute sentience we must first understand how action potentials compute in temporal space within the brain neural network (BNN). These computational mechanisms are traditionally described by the action potential (Hodgkin and Huxley, 1952). The Hodgkin Huxley equation describes the potential across the membrane of a neuron in terms of ion exchange changing over a period of time. The timing of the charging and thus the speed of propagation is defined by Cable Theory. It was assumed in 1952 that excitable membranes contained sufficient ion channels close enough together that the spread of charge from one channel could affect another. We now know this is not the case and an alternative method of propagation must be taking place to account for the speed of propagation. A problem is the lack of knowledge about the fundamental and computational mechanisms that underlie the generation and propagation of action potentials in single neurons and neuronal networks. A mechanical pulse known as a soliton always travels with the action potential at the same speed which has been considered ancillary, in this paper we show that it is this pulse that defines the speed and thus the computational mechanisms that form the basis of behavior.

Neurons are diverse and have many shapes, sizes and functions (Bullock et al., 1977). They may have evolved from secretory cells in the early metazoa. We can envisage that as animal size increased the action potential evolved to control secretions at a distance (Grundfest, 1959; Winlow, 1990) although many local circuit neurons in both vertebrates and invertebrates do not conduct nerve impulses (Dowling, 1975, 1992, chapter 13; Shepherd, 1975, 1988, chapter 10; Roberts and Bush, 1981). However, the discovery of the nature of the action potential, which is used to signal over distance, was critical to the development of modern neurophysiology. Unfortunately it has been modeled as a binary event in computational brain networks (Johnson and Winlow, 2017b). We believe this assumption to have been unnecessary and to be the wrong premise for computation both within nervous systems and in the development of artificial intelligence (AI). Furthermore, the advantages of ternary computing over binary computation are that it requires less hardware and contains more information in a shorter code. Phase ternary computing results from phase addition of a ternary pulse. Here we discuss evidence that the action potential is a temporal compound ternary structure, described as the computational action potential (CAP).

The CAP contains the refractory period, an analog third phase capable of phase-ternary computation via colliding action potentials. This would best fit a realistic BNN and provides a plausible mechanism to explain transmission, in preference to Cable Theory. The action potential pulse (APPulse), is made up of the action potential combined with a coupled synchronized soliton pressure pulse in the cell membrane. We describe a model of an ion channel in a membrane where a soliton deforms the channel sufficiently to destroy the electrostatic insulation thereby instigating a mechanical contraction across the membrane by electrostatic forces. Such a contraction has the effect of redistributing the force lengthways thereby increasing the volume of the ion channel in the membrane. Na ions, once attracted to the interior, balance the forces and the channel reforms to its original shape. A refractory period then occurs until the Na ions diffuse from the adjacent interior space.

Finally, a computational model of the action potential (the CAP) is proposed with single action potentials significantly including the refractory period as a computational element and capable of computation between colliding action potentials.

## Modeling the Action Potential

### A Lesson From Cephalopods

It is appropriate to this report that the physiology of action potentials was first modeled using the giant axon of the squid, *Loligo forbesi* (Hodgkin and Huxley, 1952). This model predicted the ionic currents crossing cell membranes to create a potential difference and changing over time due to the modulation of currents. However, one of the major problems in AI is how to code accurately for the action potential. Action potentials are critical to the operation of the brain and computation and timing of the action potential is important in considering any possible computational requirements. Thus, the mechanisms that define the speed of the action potential and its temporal accuracy will directly affect the methods of reliable computation available to the neural network. Thus changes in accuracy of action potential timing would make any form of computation unreliable.

The action potential can be divided into three computational phases, resting, threshold and refractory, the specific details of which are discussed elsewhere (Johnson and Winlow, 2017a). The first two phases may be modeled digitally, while the refractory phase is an analog event. Thus the action potential can be considered to be a phase ternary event. Phase ternary computation is an unexplored field in computation.

Action potentials travel at a speed commensurate with the membrane dynamics of the axon and have been shown to be accurate to at least 1 millisecond over its length in small neurons (Diesmann et al., 1999). The transmission dynamic of any axon or part of an axon may be different depending upon the membrane components such as the ion channel spacing (Hodgkin, 1975; Holden and Yoda, 1981; Hille, 1992) and the physical formation of the membrane.

### The Macroscopic Point of View

Measurements of the action potential are taken from both sides of the membrane and measure the potential difference across a wide area reflecting the measurement of the H&H model (Hodgkin and Huxley, 1952). An action potential travels not through the cytoplasm – where it is measured with intracellular microelectrodes – but is a product of the ion changes at the surface of the membrane. Small diameter axons (0.2 μm) have ion channels widely spread with low concentrations of ion channels (Holden and Yoda, 1981; Hille, 1992; Marban et al., 1998). All measured action potentials have been recorded at some distance from the membrane. As the action potential progresses, the micro-pipette measures current not from a point on the membrane, but from an area including multiple ion channels, and may not reflect the mechanisms of propagation from a single point. The same is true for the loose patch clamp method, where rather large (15–30 μm) (Marrero and Lemos, 2007) external patch electrodes are used and are unable to measure changes at a single point (Walz, 2007).

#### The Hodgkin Huxley Equation and Cable Theory

The H&H equation describes ionic flow across the membrane in mathematical terms over the period when the membrane reaches threshold until the end of the refractory period. The membrane can be considered polarized immediately the threshold takes effect, as after that point there is no return. After threshold the membrane is in the refractory state as no further action potential can be created. The action potential has a maximum speed of about 1 ms^-1^ in unmyelinated the axons (Waxman and Bennett, 1972). Thus, there is a ‘leading edge’ just before depolarization and as the action potential is self-propagating this leading edge must have the innate property of self-propagation. In effect it also instigates the refractory period once the depolarizing wave has passed. Patch clamping on single ion channels has demonstrated that the existence of threshold, spike and refractory period can be explained by modulation of the Na^+^ channel alone (Holden and Yoda, 1981; Marban et al., 1998; Catterall, 2012). Thus the physical origins of the potential changes associated with the action potential can be directly attributed to the ion channel mechanisms, with the activity of the sodium channels defining its progression. This implies that the only element that is responsible for the live propagation of an action potential is whatever mechanism causes the threshold at the leading edge.

#### The Threshold Alone Is the Initiator of the Action Potential

**FIGURE 1:**
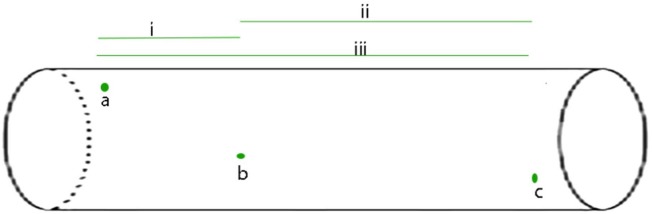
The APPulse - an illustrative, uniform axon containing three widely spaced ion protein channels is shown. For the benefit of a clear description the axon has been standardized as follows: 1 the axon is uniform such that speed along the axon by the lipid pulse is constant; 2 - the protein channels (in green) are gates that reach threshold at a voltage of V and produce a digit of Entropy E; 3 - in this axon there are no lipid channels or other proteins except the three ion channel proteins, a, b, c. The diagram illustrates the events that take place for the APPulse to continue between ion channels. On depolarization at a, an action potential digit of entropy E is created. A Lipid pulse is subsequently created by Entropy E that continues along the axon. Entropy loss e from E causes a proportionate decrease in amplitude but the velocity of the soliton is constant where the membrane components are uniform. This causes the entropy to decrease by dissipation over distance d such that Ed(b) = E-e. This residual entropy Ed (b) is enough to mechanically distort the ion channel at b breaking the electrostatic insulation. Contraction of the ion channel ensues completing the mechanism. In this model the entropy of electrostatic forces produced by the ion channel at (a) must be sufficient to produce a lipid pulse of such entropy E to arrive at (b) with sufficient entropy to break the electrostatic insulation of (b) and repeat to c, where the process again repeats itself. In smaller diameter neurons dissipation will be greater due to entropy being a function of membrane area. i, ii, and iii represent different timings between separated ion channels along the membrane. From Johnson (2015) – reproduced under the Creative Commons License.

**FIGURE 2:**
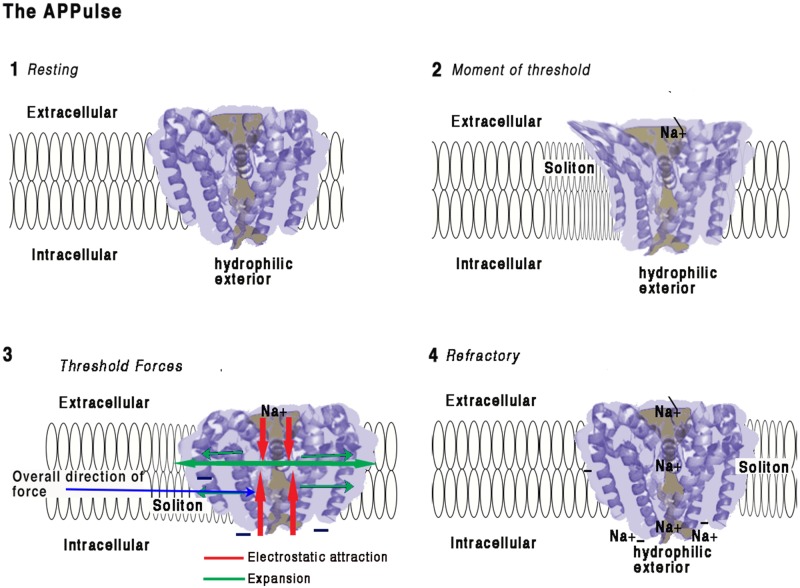
Illustration of how force from lipid may act on a eukaryotic voltage-gated sodium ion channel from the American cockroach (adapted from McCusker et al., 2012 and Shen et al., 2017).**(1) Resting:**Ion channel embedded in a membrane at resting state. The main structure of a channel is four coiled helices. One of these helices is connected to a hydrophilic negatively charged spiral placed intracellularly. The structures of the four main spirals are loosely bonded with the intracellular surface of the structure and are hydrophilic. The intracellular portion of the structure is negatively charged and polarized toward the hydrophilic spiral.Any proximate connection between extracellular positive charges and the negative hydrophilic portion will have the electrostatic effect of attraction thus drawing the intracellular side of the structure toward the extracellular surface and deforming the structure. The ion channel is surrounded by a membrane approximately 10 nm thick with the hydrophilic spiral toward the interior of the cell. The pore is effectively formed as a valve blocking ions and importantly insulating against charge, thus preventing electrostatic interaction. Any opening of the pore therefore causes entry Na ions and immediate availability of their charges for interaction. The ionic radius of Na is about 16 nm. The surface of the ion protein channel is hydrophilic, a vertical contraction would be expected and this is in fact what happens (McCusker et al., 2012)**(2) Moment of Threshold:**•  As a soliton approaches the ion channel pressure will develop on the sides of the ion channel displacing the helices and removing the electrostatic insulating seal. This produces immediate access for electrostatic attraction between the Na and the negative interior surfaces of the ion channel. Constriction of the central helix produces a mechanical invagination of the four helixes at the pore and simultaneously opening the pore sufficiently to destroy its electrical insulating capacity.•  Positive charges are attracted to the intracellular space but also to the hydrophilic surfaces of the pore. The Na^+^ ions are in electrostatic proximity to the negatively charged portions of the structure the moment the electrostatic insulation is broken and are drawn inward.**(3) Threshold Forces:**The incoming Na^+^ ions are electrostatically bound to the selector causing an opposing force to the hydrophilic parts of the structure which causes the s6 spiral (for detail see Shen et al., 2017) to be drawn toward the Na^+^ receptor. Movement of the hydrophilic s6 portion will undo the main spiral causing the pore to open mechanically in an iris movement (McCusker et al., 2012), which causes a mechanical contraction of the structure. At threshold, the action of contracting the structure from external to internal during pore opening creates forces along the surface of the membrane as the helices are pressured outward. This action is synchronized to the arrival of the soliton and the mechanical energy will be transferred to the soliton, thereby reducing entropy.**(4) Refractory:**When sufficient positive charges have reached the interior equalizing charge adjacent to the hydrophilic spiral, the forces equalize and the pore will close. The pore and structure of the ion protein channel is now at rest. There is now a large concentration of Na^+^ ions in the vicinity of the hydrophilic regions and the activating spiral. Any further soliton or disturbance to the membrane will not cause activation of the ion channel pump until the excess Na^+^ ions are removed by diffusion. The ion channel is refractory until this charge is cleared allowing for further electrostatic attraction. Synchronization is achieved between the ion channel pump and the soliton and electrostatic force is transferred to mechanical force and then to the soliton.This work is a derivative of Figure 2A,D | Crystal structure of the NavMs pore by McCusker et al. (2012), used under Creative Commons BY-NC-SA 3.0. This work is licensed under CC BY-NC-SA 4.0 by Andrew Johnson and William Winlow.

It is also the rate limiter to the velocity of the action potential along the axon. The timing of the spike is therefore directly related only to the threshold. The threshold may be better defined temporally so that it is not a potential difference but a change over time in terms of action at the level of the membrane, i.e., the equilibrium point at which each subsequent ion channel opens. The predominantly voltage gated Na^+^ channels (Yu and Catterall, 2003) open when positive charge approaches their s4 units. This all-or-none point is assumed to be the point of threshold. Conventionally, in the H&H model, threshold is assumed to be a direct result of Na ions arriving from a neighboring channel. For the APPulse structure **Figure 1**, the mechanical pulse opens the channel enough for the electrostatic insulation to break causing electrostatic attraction between the positive Na^+^ ions passing through the s4 units and the negative parts of the ion gate structure. A mechanical contraction occurs transferring entropy (energy dispersal) directly to the soliton. Na^+^ ions continue to be attracted to the negative intracellular space until the negative charge is balanced, at which point the gate is closed. The remaining positive ions adjacent to the negative regions of the ion channel prevent any further opening, and remain deactivated until the Na^+^ charges diffuse into the intracellular space. This is explained in **Figure 2**, where a voltage–gated Na^+^ channel is illustrated. The ion channel is therefore reactive both to mechanical and charge stimulus. Entropy from the soliton is only required to break the electrostatic-insulation of the ion channel for membrane contraction to occur, thus producing the next entropy charge for the soliton to proceed. The threshold is defined therefore as the time for the s4 units to fully open the channels. The rest of the action potential is only concerned with the refractory period and stabilization to resting potential. It is irrelevant to speed of transmission, although of course the refractory period can affect the frequency of transmission.

### The Microscopic Point of View

At a microscopic level the mechanism of the threshold can be investigated knowing the properties of just the Na^+^ channels and the membrane, for example using the patch clamp technique. The Na^+^ channel has been isolated and its properties understood (Catterall, 2013; Shen et al., 2017). In addition, the speed and flow of an action potential is directly dependent upon the speed of activation of the threshold potential corresponding to opening of the Na^+^ gates which may be achieved mechanically (see below), the resulting entropy causing a soliton. The H&H model is a proven advantageous means for the demonstration of ion transport and passage through a membrane over a large area, but because it cannot explain charge flow along the length of the membrane it must be allied with a mechanical model. However, it should be understood that because of the physical properties of Na^+^ ions, threshold charge cannot flow from one ion channel to the next in the time available. There must therefore be another mechanism present and a ‘soliton’ mechanical pulse is known to be present. This is the force-from-lipid mechanical energy (Brohawn et al., 2014) that opens the Na^+^ channels. This coupled APPulse fulfills the requirement for a continuing action potential where Cable Theory fails.

Cable Theory defines the potential arising from ion disparity across the membrane as a capacitance. Historically Cable Theory (Rall, 1962, 1995) comes from a direct analogy from capacitance theory and considers charge over the whole surface of an insulator (Poznanski, 2013). Thus, Cable Theory is a mathematical construct to predict observations on large areas of membrane, in which the depolarization of the membrane during the action potential is analogous to the discharge of a charged capacitor. Experimentally, the mathematics of cross current resistances appear to work correctly over a large part of the membrane in the macroscopic view and this is the basis for the H&H model. Cable Theory itself has recently been revisited to demonstrate that both neuronal morphology (Bestel et al., 2017) and the extracellular medium (Bédard and Destexhe, 2013, 2016) exert significant influences on neuronal cable properties as might be expected. Such effects require considerable mathematical modifications to the original theory.

When H&H were conducting their experiments accurate depictions of the membrane, ion channels and specifically inter-channel distances were not available, but were assumed to be “separated by an infinitesimal distance” (Hodgkin, 1975). It was not until single patch clamping became available (Hille, 1992) that accurate measurements of channel density became known (Hamill et al., 1981; Goodman et al., 2005). At the time that H&H described longitudinal flow, the assumption was that there was a mechanism by which activation of an ion channel during threshold would activate adjacent ion channels. This was thought to produce a cumulative and on-going propagation – just as the charging electrons distribute evenly and almost instantaneously. However the mechanism of propagation between channels was not identified then and has still not been identified. Thus, the action potential takes place at the level of the membrane, which means that the resulting observation is due to all the combined ionic changes in a very wide portion of that membrane.

Mechanisms must exist in the membrane to produce an on-going pulse. Intra-Ion channel distances have been measured accurately although this distance is variable. Single patch clamping a membrane will usually only detect a single active ion channel (or possibly two) if the pipette is sized about 1.5 μm. As the membranes examined contain many different ion channels we can confidently say they are spaced at least 1.5 μm apart. Thus “Channels are not crowded” together (Hille, 1992, pp. 334–335) and the distance over which a single Na ion can affect another channel is limited by the time taken to travel through the ion channel and to the next channel. The ionic radius of Na^+^ is about 116 pm (Shannon, 1976) indicating that a Na ion has to lie adjacent or in very close proximity to the Na^+^ channel to activate it and the channels are ion specific. The time required for charge to spread from one ion channel to the next can be calculated from the ionic radii and the diffusion coefficient (Goodman et al., 2005). A conservative simple Speed-Time calculation suggests that the maximum speed that charged Na ions can travel between channels is less than 1/1000 of what is necessary for propagation (Johnson, 2015). Cable Theory only models the ion flows of the action potential under conditions of voltage clamp, but as yet there is no known mechanism for propagation of the action potential provided by Cable Theory. Thus, the H&H model very clearly demonstrates the electrically measured activity surrounding the underlying mechanism of propagation from one channel to the next, but not the mechanism itself.

## Is There Evidence for a Mechanical Component in Action Potential Propagation?

Two supposedly incompatible models for action potential propagation have been proposed and compared (Appali et al., 2012), the H&H model and soliton theory. However evidence at the level of the membrane structure suggests the two models are compatible and are synchronized; the H&H model at the macroscopic level and the soliton model at the microscopic level. In the H&H model of the membrane, for propagation to occur positive ions would have to behave as electrons but positive charges are a part of the atom and so physically a sodium atom must move for the charge to move. Without a mechanical component the H&H action potential cannot propagate. Recent evidence suggests that a “force-from-lipid” model (Brohawn et al., 2014) could transmit pulses into mechanosensitive ion channels in the absence of other cellular components and might also explain propagation through the membrane lipid. We envisage that it would take the form of a soliton known as an APPulse which would be the precursor of ion channel opening.

### The APPulse

There is now a large body of evidence showing that:

• a ‘soliton’ mechanical pulse accompanies an action potential and is stable propagating at constant velocity (Tasaki and Iwasa, 1982; Heimburg and Jackson, 2005; El Hady and Machta, 2015)
• ion channel separation is too great to allow for ion channel interference from adjacent channels caused by ionic charge (Holden and Yoda, 1981; Marban et al., 1998; Catterall, 2012; Johnson, 2015)
• ion channels can be opened by mechanical stimulus (Martinac, 2012; Anishkina et al., 2014; Takahashi et al., 2016; Zhang et al., 2016)
• there is deformation of the membrane by activation of ion channels (Tasaki and Iwasa, 1982; El Hady and Machta, 2015)
• entropy (thermodynamic) measurements do not follow the H&H action potential but do follow the APPulse. (Abbott, 1958; Howarth, 1975; Ritchie and Keynes, 1985; Tasaki and Byrne, 1992; Moujahid et al., 2011).

The action potential measured by H&H is a measure of the sum of all the potential changes of all charges across the membrane over a wide area. The result is always a combination of effects from many ion channels, some open, some closed and some refractory. However, direct mechanical stimuli of axons can elicit action potentials (Howe et al., 1977) suggesting the involvement of a mechanical component (Appali et al., 2012).

### The Na^+^ Channel Is an Electro-Mechanical Soliton Pump

Positive ions do not behave as electrons and require time and the correct diffusion coefficient to move. Calculation of ion channel distribution from single channel studies, demonstrates that Cable Theory can only account for the action potential in its stable states (resting or maintained by voltage clamp) (Johnson, 2015). The ion channels are spread too far apart and the entropy changes do not match those predicted by the model. The longitudinal resistance in the H&H arrangement is always infinite as there is no mechanism that provides surface spread depolarization. Surface spread and thus speed of action potential must therefore occur by another mechanism. We suggest that this is by a mechanical ‘soliton’ coupled and synchronized to entropy provided by the ion exchangers described (**Figure 2**). The soliton pulse mechanically opens ion channels leading to further depolarization. Thus a soliton always occurs when a nerve impulse is generated. The membrane soliton is powered by direct mechanical forces from the opening of the pore. These forces originate from charged particles of Na attracted by the hydrophilic units and hydrophilic parts of the structure with electrostatic forces between the Na ions and the negatively charged parts of the ion channel structure. This model accounts for the threshold, spike and refractory period on its own. It produces the correct entropy/time profile if it is assumed transfer of entropy is an almost adiabatic process (in which energy is directly transferred without loss of matter or heat). Furthermore, it explains the ion changes across the membrane. Communication occurs not at the level of the action potential but at the level of ion-channel-pump to ion-channel-pump. In effect computation takes place within the membrane at the rate of transmission. Propagation of membrane pulses is well supported by Mussel and Schneider (2018) who suggest that action potentials may be better described as non-linear acoustic pulses propagating along lipid interfaces and which annihilate on collision, a well-known property of colliding action potentials (Follmann et al., 2015).

### Myelinated Fibers

This model also explains action potential transmission in a myelinated fiber where a soliton pulse created at the node of Ranvier, due to a high concentration of ion channels, is then attenuated by the rigidity of myelin. Insulating the movement of the axon membrane has the effect of reducing entropy loss and thus increases efficiency. In a small cylindrical axon, sleeved in myelin, movement is restricted and the entropy created at the nodes of Ranvier cannot be transferred along the axon membrane. Entropy is therefore directionally guided on entry into the myelin sheath creating a pulse-wave within the cytoplasm of the axon. This is similar to the action of pulse wave velocity (PWV), the velocity at which the arterial pulse propagates through the circulatory system. The mathematics is similar to the Moens–Korteweg equation (Moens, 1878) that states that PWV is proportional to the square root of the incremental elastic modulus of the vessel wall given a constant ratio of wall thickness – the myelin sheath. On exiting the myelin sheath, entropy will be transferred back to the axon membrane restarting the APPulse.

### Unmyelinated Fibers

The APPulse velocity is a result of the factors contributing to the fluid dynamic qualities of the axon membrane at any point. However, if there are insufficient ion channels producing insufficient energy, then the pulse will not reach subsequent channels and will fail. Thus, in unmyelinated fibers, the action potential travels at a speed commensurate with the membrane dynamics in each part of the axon or neurite. Speed of axonal transmission, and therefore the time impulses take to reach their destination, is variable and depends upon the axon type and diameter (**Figure 2**). Thus, the action potential as expressed by H&H is a measurement of the progression of ionic charge over the axon membrane, it cannot represent the mechanism of propagation.

## Computation by Phase Diffraction

**FIGURE 3:**
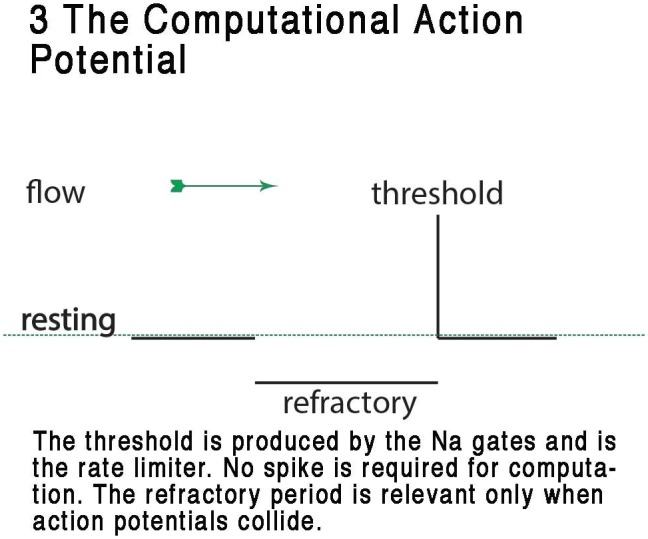
Representation of the CAP, showing the digital resting phase, digital threshold and the analog refractory phase. In this view, the resting potential serves as the ternary 0 of a ternary action potential. Any rise above threshold is the digit or +1 and the refractory period acts as phase -1 refracting any digit+1 during collision to 0. The refractory period is analog and is a result of the specific dynamic of the membrane at that point where the action potential exists. From Johnson and Winlow (2017a) – reproduced under the Creative Commons License.

In both vertebrates and invertebrates many neurons are multibranched and some have more than one spike initiation zone (Haydon and Winlow, 1982; Ledergerber and Larkum, 2012). This indicates that back-propagation of action potentials (Stuart and Sakman, 1994) can occur under natural circumstances, allowing action potentials to collide. Deconstruction of the computational variables that can be attributable to the action potential is shown in **Figure 3**: the CAP. Examination of the CAP reveals an inherent ability to compute in a realistic artificial BNN by action of the analog time component of the refractory phase, which is effectively able to reroute (diffract) action potentials along different pathways through the neural network; i.e., the refractory period is capable of interference at axon bifurcations and the axon hillock of cell bodies to produce effective deflection of action potentials along different axonal pathways in the neural network or to cause mutual occlusion.

**FIGURE 4:**
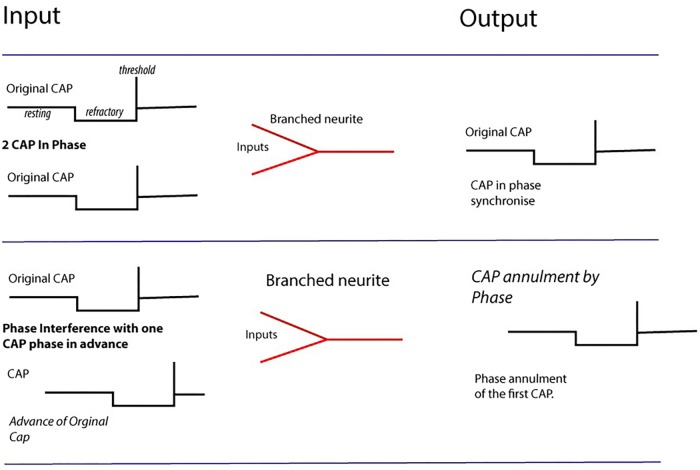
Computation by phase ternary interactions – Collisions from CAPs may result in nullification of CAP (second row) depending upon phase and the dynamics of the membrane at the point of collision. Multiple collisions form patterns navigating pathways through the network. Action potentials firing in-phase map together, whilst those with +1 and –1t overlapping cancel to 0.

Annihilation will occur between two action potentials if the refractory period of one action potential interferes with the threshold potential of another (Follmann et al., 2015; Berg et al., 2017; Johnson and Winlow, 2017a). The result of this is that as parallel action potentials meet within a neural network they diffract at branching or junctional points and may be diverted along new pathways. This diffraction occurs due to the timing arrival of the refractory phase in from the point of entry of the action potential into the neural network. The action potential then enters the neural network with a specific refractory period on a timescale defined by the membrane dynamics at that point (**Figure 4**). As the action potential flows along the neurites to the soma it may, at branching points, encounter other action potentials from parallel entry points similarly identifiable by refractory period.

**FIGURE 5:**
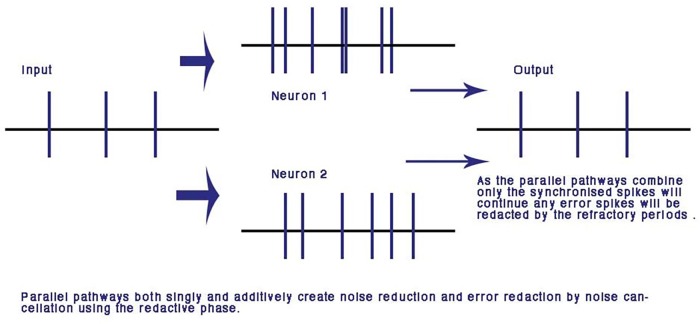
Error and noise redaction by phase ternary computation. In parallel connections, any out of phase errors and spontaneous spikes are automatically redacted by the refractory periods of neighboring spikes: using the CAP any phase shift or spontaneous spike will be reduced to the resting potential by the colliding neighbor CAP. From Johnson and Winlow (2017a) – reproduced under the Creative Commons License.

A major advantage of computation with the refractory phase is that along parallel pathways with similar dynamic transmission timing, error is redacted and the pulses synchronize. Over many iterations of nodes this makes the network error and noise free (Johnson and Winlow, 2017a). This is unique to the phase ternary computation and is the first time an error redaction mechanism has been described for a physiological neural network. Error redaction is critical in the smaller neurites of the neural network where signal to noise ratios are of the same order of magnitude as each other (**Figure 5**).

## Computation by Threshold and Refractory Period

The threshold is the point at which, either by mechanical (Johnson, 2015) or electrical means (Hodgkin and Huxley, 1952), the Na^+^ gates open at a distinct site along the axon membrane. The accuracy of timing in the APPulse can be thought of as the time taken for the threshold to pass between adjacent Na^+^ ion channels small neurons where ion channels are separated along the axon as determined by the velocity of the action potential. This distance is from 1 to 5 μm corresponding to a maximum temporal accuracy of about 1 μs if an action potential is traveling at 1 ms^-1^. The figure of 1 μs can be thought of as the maximum point of accuracy for computation and is a variable figure according to the dynamics of the membrane.

### Inhibition of Incoming Signals by Phase Ternary Annulment

Given that the ternary analog refractory phase often far outlasts the action potential, it is clear that synaptic inputs to a neuron will be obliterated during the refractory period, although powerful inhibitory postsynaptic potentials could prolong it, particularly if delivered during the relative refractory period. The refractory period is a result of protein formation changes and its duration is highly variable. In CAP dependent computation it is the refractory period (defined by the transmission dynamics of the point of interaction, and thus a non-linear time variable) that defines computation. In both the H&H model and the APPulse, the ion channels are inactivated during the refractory period. The refractory period cancels adjacent, out of phase, action potentials when they collide annihilating one of them to produce a change in phase. A neuron is thus capable of taking distinct all-or-none action potential inputs in specific temporal phase and, by interference, changing the phase to create distinct all-or-none action potential outputs. Each neuron is therefore capable of fulfilling computation independently.

## Noise Reduction

The usefulness of artificial-networks to model activity is limited by the amount of noise and spontaneous activity in biological systems and synaptic studies give little insight into conduction in more highly evolved neural-networks, where axon conduction is diverse and seemingly unreliable with an alarming amount of noise (Bullock, 1958), which must be reduced for any neural network of depth. The parallel processing of axons leading to the same neuron reduces variability in the CAP neural network where connections are randomly formed and neurons are connected by more than one pathway and may reduce signal to noise ratio (**Figure 5**). In a concatenated balanced phase system with many interfering CAPs, noise is automatically negated at each parallel point of processing by interference. Error redaction occurs where pulse trains travel along adjacent parallel pathways whether through one or more neurons or points of contact. This is a mathematical certainty where two CAPs collide from parallel pathways to a common node the trailing refractory period will annul the second threshold. This error negation is particular to phase ternary and depends upon the logic of interference where two spikes are compared from different sources and tested automatically for a match. Error in this system is negated at the point of balanced phase computation and activity within the network becomes synchronized.

## Conclusion

• We have combined the Hodgkin-Huxley model of the action potential with the soliton theory to produce a unified model of action potential propagation, the APPulse, which also applies to cardiac action potentials. This model is not restricted to spiking neurites and can be applied to non-spiking neurons and any active membrane consisting of ion channels.
• The Hodgkin Huxley model informs only the level of progression at the macroscopic level, but not the underlying mechanical processes. In actuality action potential propagation is due to a combination of separate *microscopic* elements: the APPulse, an electromechanical mechanism incorporating a soliton-ion channel pump, which produces a phase ternary CAP from the separately identifiable resting, threshold and refractive phases. Recovery occurs when the Na^+^, K^+^ and other ions re-establish membrane stability.
• Phase-ternary computation within physiological neural networks is fast, accurate to microseconds, and efficient. It diffracts parallel inputs within a network along pathways defined by the phase in which action potentials arrive at the neural network in temporal synchrony. In contemporary computing parlance: phase-ternary computation is the brain’s machine language and is capable of storing information regardless of any other memory storage or retrieval processes within that network.
• In the H&H action potential the temporal accuracy of the point of computation is variable, restricted to accuracy estimated from the H&H curve. Computation is only accurate to calculated milliseconds. By contrast computation with the action potential pulse is accurate to the exact threshold distance between specific ion channels in microseconds along an unrestricted neurite giving 1000 times greater computational precision.

## Author Contributions

AJ: the original concept. AJ and WW: split the writing of the review about 50/50 with much discussion between us about the way to finalize the article.

## Conflict of Interest Statement

The authors declare that the research was conducted in the absence of any commercial or financial relationships that could be construed as a potential conflict of interest.
